# Anti-Inflammatory and Anti-Oxidant Potential of the Root Extract and Constituents of *Doronicum austriacum*

**DOI:** 10.3390/molecules22061003

**Published:** 2017-06-16

**Authors:** Stefania Marzocco, Simona Adesso, Mostafa Alilou, Hermann Stuppner, Stefan Schwaiger

**Affiliations:** 1 Department of Pharmacy, University of Salerno, Via Giovanni Paolo II 132, I-84084 Fisciano SA, Italy; smarzocco@unisa.it (S.M.); sadesso@unisa.it (S.A.); 2 PhD Program in Drug Discovery and Development, University of Salerno, Via Giovanni Paolo II 132, I-84084 Fisciano SA, Italy; 3 Institute of Pharmacy, Pharmacognosy, Member of the CMBI, University of Innsbruck, CCB, Innrain 80-82, 6020 Innsbruck, Austria; mostafa.alilou@student.uibk.ac.at (M.A.); hermann.stuppner@uibk.ac.at (H.S.)

**Keywords:** *Doronicum austriacum*, benzofurans, macrophages, astrocytes, inflammation, oxidative stress, NO, ROS, nitrotyrosine

## Abstract

Background: *Doronicum austriacum* Jacq., Asteraceae, is a plant which is used in traditional alpine medicine. Historical sources describe the medical use of the root, but up until now only a few studies evaluated its pharmacological properties. The evaluation of the dichloromethane extract, and its major compounds for their anti-inflammatory and anti-oxidant potential was performed in macrophages J774A.1 and C6 astrocytes. Nitric oxide (NO) and reactive oxygen species (ROS) release, as well as nitrotyrosine formation, were evaluated. Moreover, in order to evaluate the potential anti-proliferative activity, under the same experimental conditions, 3-(4,5-dimethyltiazol-2yl)-2,5-phenyl-2*H*-tetrazolium bromide (MTT) assay was also performed. Our results indicate that *Doronicum austriacum* has a significant effect in inhibiting both pro-inflammatory and pro-oxidative mediators. All isolated compounds were able to significantly inhibit NO and ROS release both in macrophage and in astrocytes cells, even if the effect was more pronounced in macrophage. In particular, among the tested compounds, 6,12-dihydroxy-(−)-2*S*-tremetone exerted stronger activity. Both extract and single compounds did not affect cellular viability. This study provides evidence for the pharmacological anti-inflammatory and anti-oxidant potential of *Doronicum austriacum* extract. These effects could be due to the activity of its major constituents and subsequent identification of benzofurans as a promising compound class to combat inflammation and related diseases.

## 1. Introduction

Inflammation is a response triggered by tissue injury, as well as by microbial infection, and it can lead to the risk of sepsis, multiple organ failure, and death. Inflammation is necessary to eliminate aggressors, but it can be detrimental if the initial reaction is not limited. In these cases, anti-inflammatory compounds are administered to control this response and are therapeutically useful. Macrophages play a pivotal role in defending the host during the inflammatory response. However, their excessive activation may contribute to extensive tissue damage. The activation of these cells by pro-inflammatory stimuli, such as lipopolysaccharide (LPS) from gram-negative bacteria like *E. coli*, induces the production of large amounts of mediators involved in the inflammatory onset such as nitric oxide (NO), pro-inflammatory enzymes, and cytokines [1]. Reactive oxygen species (ROS) regulate many physiological responses in human health. Like inflammation, if not properly regulated, the oxidative response could also lead to a number of harmful effects mediating many aspects of inflammatory-induced tissue damage and dysfunctions [2,3]. Oxidative stress and neuro-inflammation also contribute to the pathogenesis of neuronal degeneration [4] and can cause cell membrane damage from lipid peroxidation, changes in protein structure and function due to protein oxidation, and structural DNA damage hallmarks of several neurodegenerative diseases [5,6,7,8]. The central nervous system is particularly vulnerable to oxidative stress, possibly due to its high oxygen demand, polyunsaturated fatty acids, and low levels of glutathione (GSH) [9,10]. Increasing ROS production can exacerbate the expression of inflammatory mediators as has been detected in patients with neurodegenerative disease [11]. Nowadays, considering the role of inflammation and oxidative stress in many chronic diseases, the study of natural compounds with anti-inflammatory and anti-oxidant potential has been of growing interest among scientists. Positive results of an in-house screening showing nitric oxide (NO) inhibitory activity of the dichloromethane (DCM) extract of the roots of *D. austriacum* prompted a detailed phytochemical and pharmacological (re)investigation [12]. *Doronicum austriacum* Jacq., Asteraceae, is a plant which is used in traditional alpine medicine. Historical sources describe the use of root extracts as a vermicidal and laxative agent, but also in heart strengthening preparation [13]. Up to now, only a few pharmacological studies [14] have evaluated the pharmacological properties of *Doronicum* extracts. One described activity is a cytostatic effect on mice fibroblasts by a currently not further characterized alkaloid enriched fraction (positive reaction on TLC with Dragendorff-, Diazo- and modified Ehrlichs-reagent) of the roots of *D. austriacum*. Due to the positive chemical reactions, the alkaloid fraction was suspected to consist of pyrrolizidine alkaloids, which are widely distributed in the sunflower family.

## 2. Results

### 2.1. Identification of the Major Compounds

**Figure 1:**
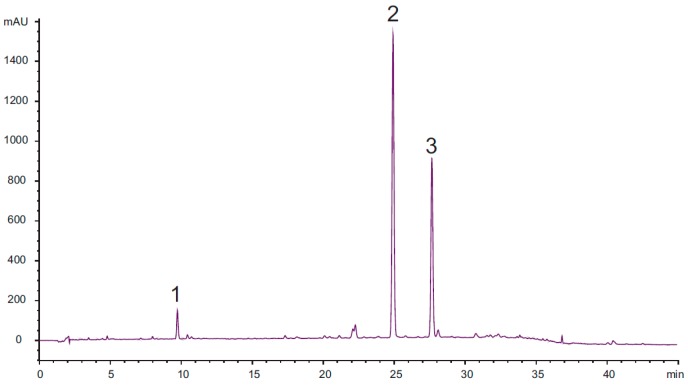
Chromatogramm of the HPLC-DAD analysis of the investigated dichloromethane (DCM) extract of the roots of *Doronicum austriacum* (2.5 mg/mL MeOH) at 205 nm. Analytical conditions: stationary phase: Phenomenex Synergi Max-RP 80 Å, 4 µm (4.6 mm × 150 mm); temperature: 35 °C; mobile phase: A = water + 0.025% TFA, B = acetonitrile; flow rate: 1.00 mL/min; detection: 205 nm; injection volume: 10 µL; solvent composition during analysis: 0′: 75% A; 10′: 60% A; 30′: 30% A; 35′: 2% A; 45′: stop; post time: 10′.

HPLC analysis of the DCM extract of the roots of *Doronicum austriacum* showed the presence of three major compounds when monitored at 205 nm (Figure 1). 

**Figure 2:**
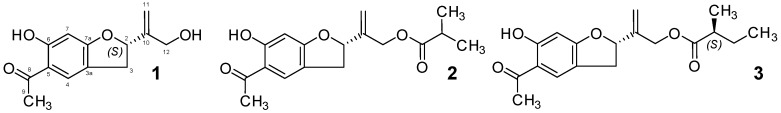
Chemical structure of compounds **1** to **3**.

Isolation and structure elucidation by LC-MS, 1- and 2D-NMR spectroscopy and optical activity determination enabled the identification of compound **1** as 6,12-dihydroxy-(−)-2*S*-tremetone, compound **2** as (*S*)-2-(5-acetyl-6-hydroxy-2,3-dihydrobenzofuran-2-yl)allyl isobutyrate, and compound **3** as 2-((*S*)-5-acetyl-6-hydroxy-2,3-dihydrobenzofuran-2-yl)allyl (*S*)-2-methylbutanoate (see Figure 2). Although the root material was already investigated in 1970 [12], only derivatives bearing an 12-hydroxy-, or an 4,12-dihydroxy-benzofuran skeleton, were identified. **1**–**3** are described in a later published work dealing with constituents of *D. pradalianches* [15], incorrectly stating that those were already isolated from *D. austriacum* [12]. A third manuscript of the same authors describing the investigation of *D. marcrophyllum* [16] contains for the first time ^1^H-NMR-data of **3**. Since no further NMR data of compounds **1**–**3** are published, the obtained values are summarized in Table 1.

**Figure 3:**
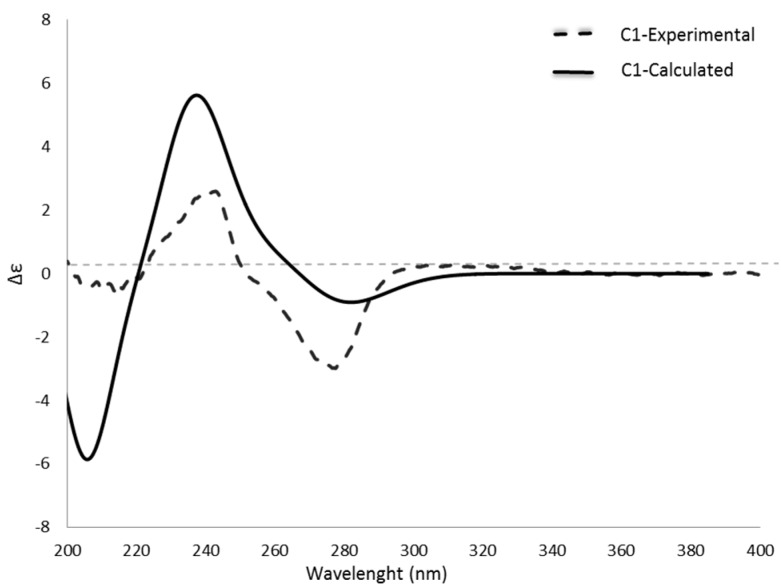
Measured (acetonitrile) and calculated ECD-spectra of compound **1**.

The stereochemistry of the benzofurane skeleton was predictable by the measured optical rotation: **1**, ${\left[\alpha \right]}_{20}^{D}$: −8.06 (*c* 0.124, MeOH); **2**, ${\left[\alpha \right]}_{20}^{D}$: −21.96 (*c* 0.970, MeOH); **3**, ${\left[\alpha \right]}_{20}^{D}$: −4.76 (*c* 0.124, MeOH) and comparison with literature values of related compounds [17]. In order to prove this hypothesis, a comparison of calculated and experimental ECD spectra was performed. Since the experimental spectra (see Figure 3; Figures S11 and S17) showed no differences, except some noise in the lower wavelengths (compound **2**, see Figure S11) due to the presence of minor impurities, an identical absolute configuration at C-2 of all three compounds was assumed. Although compound **3** comprises an additional chiral center adjacent to a weak chromophore-carbonyl group, the transitions of this carbonyl group was found to be obscured by the strong chromophore (benzene ring) present in the analyzed scaffold. In order to reduce the calculation time, the structure of compound **1** was selected as a representative for further calculations.

Therefore, the 3D structure of **1** was subjected to a conformational analysis in H_2_O by Optimized Potential for Liquid Simulations (OPLS-3) force filed. Conformers in an energy window of 2 Kcal/mol were selected and geometrical optimization in gas phase (DFT/B3LYP/6-31G**) was performed. Calculation of free energies indicated the presence of eight conformers of compound **1** (see Figure S18). The calculated ECD spectrum of compound **1** was in good agreement with the experimental spectrum especially CEs at 275 nm, 240 nm, and 215 nm (see Figure 3), while the corresponding enantiomer showed the opposite ECD (not shown). From this, absolute configuration of C-2 in the isolated three compounds was established as *S*. The absolute configuration of the second chiral center of compound **3** was determined by a hydrolysis experiment, which showed a positive optical rotation for the free acid which enabled its identification as (*S*)-(+)-2-methylbutyric acid.

### 2.2. Anti-Inflammatory and Anti-Oxidant Potential of the D. austriacum Extract and Isolated Compounds in J774A.1 Macrophages and in C6 Astrocytes

**Figure 4:**
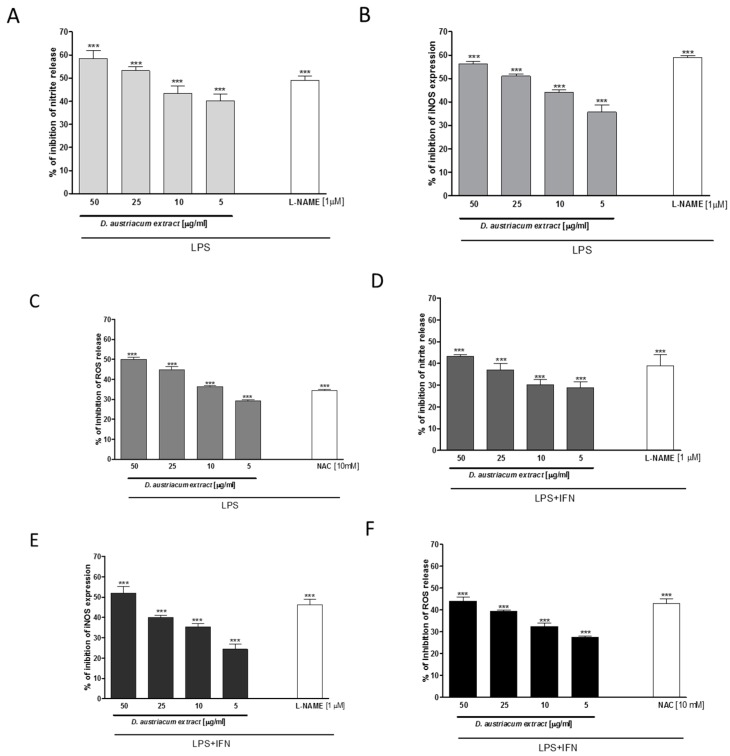
Effect of *Doronicum austriacum* DCM root extract on nitrite release, index of NO production (**A**,**D**), measured by Griess reaction, iNOS expression, evaluated by cytofluorimetry (**B**,**E**), and on ROS production (**C**,**F**), measured by DCHF assay, in J774A.1 macrophages (**A**–**C**) and C6 cells (**D**–**F**). Data are expressed as mean ± S.E.M.; *** denotes *p* < 0.001 vs. LPS (1 μg/mL) or LPS + IFN-γ (1 μg/mL; 100 U/mL). L-NAME (1 μM) was used as positive control for inhibition of nitrite release and iNOS expression and inhibited nitrite production with a percentage of 49.00 ± 2.08% and with a percentage of 59.00 ± 0.74% iNOS expression in J774A.1 macrophages. In C6 cells, L-NAME inhibited nitrite release with a percentage of 39.33 ± 4.37% and iNOS expression with a percentage of 46.21 ± 2.65%. *N*-acetyl cysteine (NAC; 10 mM) was used as positive control for ROS release inhibition, and gave a percentage of inhibition of ROS production of 34.50 ± 0.50% in J774A.1 and of 43.33 ± 1.45% in C6 cells, respectively.

In macrophages, LPS triggers an inflammatory response that culminates in the release of pro-inflammatory mediators. NO is considered a pleiotropic mediator that acts in a variety of physiological and pathophysiological processes. When macrophages are activated by LPS, massive amounts of NO are produced to exert a nonspecific immune response, from inducible nitric oxide synthase (iNOS). Induced NO is a “final common mediator” of inflammation, but is also essential for the up-regulation of the inflammatory response. In our experiments, a dichloromethane root extract (5–50 µg/mL) of *D. austriacum* significantly reduced NO release (*p* < 0.001 vs. LPS alone; Figure 4A), and iNOS expression in macrophages at all tested concentrations (*p* < 0.001 vs. LPS alone; Figure 4B).

ROS, as well as NO, generation in the inflammatory site is typically induced as part of a defensive reaction intended to clear infectious and environmental threats, including microbial agents and particulate material. Alternatively, ROS activation could act as a significant and adverse participant in inflammatory disease. LPS (1 μg/mL) induced a significant ROS production in macrophages after 24 h. Treatment with the extract, at all tested concentrations (5–50 µg/mL), significantly, and in a concentration-related manner, reduced ROS production in macrophages (*p* < 0.001 vs. LPS alone; Figure 4C), thus also indicating its antioxidant effects. Neurodegenerative diseases are considered a growing group of disorders, and evidence indicates that neuroinflammation and oxidative stress contribute to pathogenesis in these diseases. Therefore, the anti-inflammatory and anti-oxidant potential of the root extract (5–50 µg/mL) was evaluated for the impact on NO release, iNOS expression, and ROS levels in LPS + IFN-γ stimulated astrocytes primarily involved in central nervous system (CNS) homeostasis [18]. Our results indicate that the *D. austriacum* root extract significantly, and in a concentration-related manner, inhibited NO release, iNOS expression, and ROS production in C6 astrocytes, at all tested concentrations (*p* < 0.001 vs. LPS alone; Figure 4D–F).

**Figure 5:**
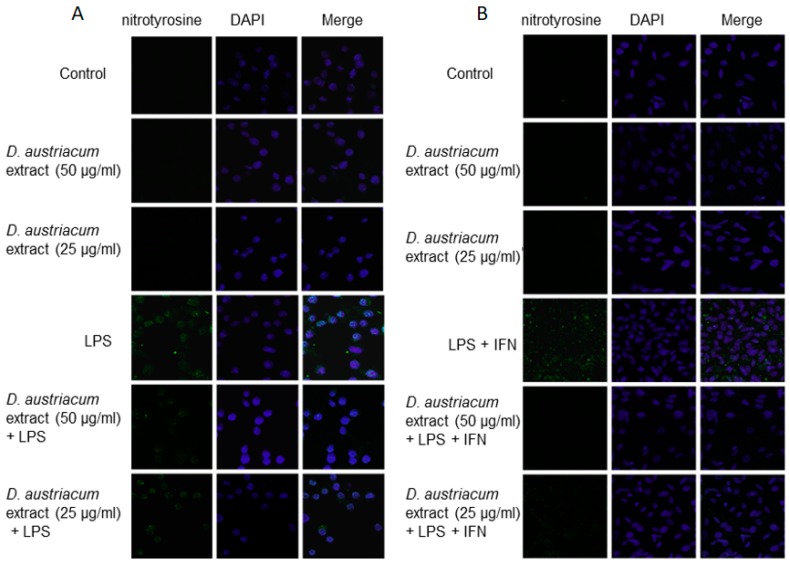
Effect of *Doronicum austriacum* DCM root extract (25 and 50 µg/mL) on nitrotyrosine formation evaluated by confocal microscopy in J774A.1 macrophages (**A**) and C6 astrocytes (**B**) alone, or in combination with LPS (1 μg/mL) or LPS + IFN-γ (1 μg/mL; 100 U/mL) after 24 h.

NO produced during inflammation also rapidly reacts with superoxide anion generating the toxic metabolite peroxynitrite. Peroxynitrite is able to nitrate tyrosine residues in proteins, resulting in the formation of nitrotyrosine. Because nitration of tyrosine is an alternative to phosphorylation at key residues, it can affect a protein’s enzymatic activity and interfere with intracellular signaling processes [19]. By immunofluorescence assay, we are able to show that *D. austriacum* root extract (25 and 50 µg/mL) alone does not induce nitrotyrosine formation in both J774A.1 and C6 cells with respect to cell control (Figure 5A,B). LPS or LPS + IFN-γ added to macrophages and astrocytes respectively induced an increase of nitrotyrosine formation. When *D. austriacum* root extract (25 and 50 µg/mL) was added 1 h before and simultaneously with LPS or LPS + IFN-γ followed by an incubation for 24 h, a reduction of nitrotyrosine formation was observed in both cellular lines (Figure 5A,B). This suggests that *D. austriacum* root extract could reduce nitrotyrosine formation in both macrophages and astrocytes cells.

**Figure 6:**
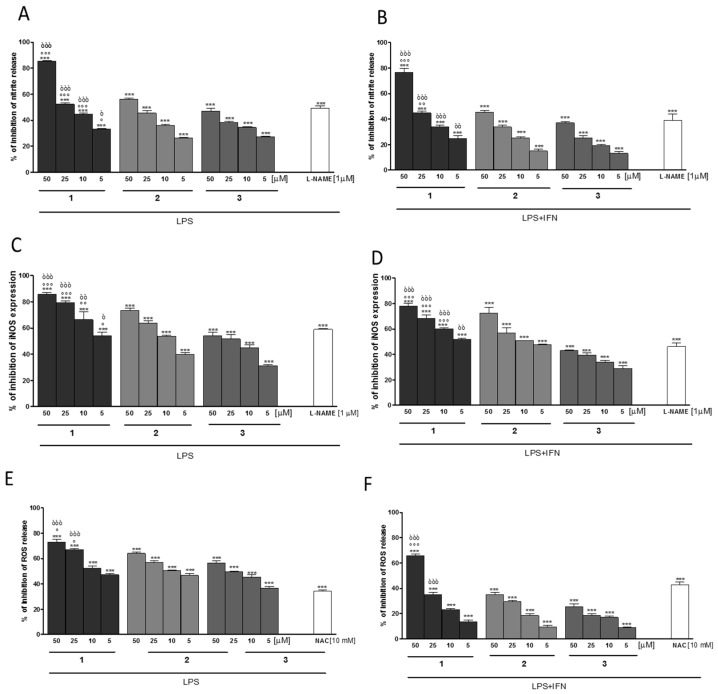
Effect of compounds **1**–**3** (5–50 µM) on nitrite release (**A**,**B**), iNOS expression (**C**,**D**), and on ROS production (**E**,**F**), in J774A.1 macrophages (**A**,**C**,**E**) and C6 astrocytes (**B**,**D**,**F**) after 24 h. Data are expressed as mean ± S.E.M.; *** denotes *p* < 0.001 vs. LPS (1 μg/mL) or LPS + IFN-γ (1 μg/mL; 100 U/mL). °°°, °°, ° denote *p* < 0.001, *p* < 0.01, *p* < 0.05 vs. **2**. òòò, òò, ò denote *p* < 0.001, *p* < 0.01, *p* < 0.05 vs. **3**. L-NAME (1 μM), used as positive control for NO and iNOS inhibition gave a percentage of inhibition of 49.00 ± 2.08% for nitrite production of and a percentage of inhibition of 59.00 ± 0.74% of iNOS expression in J774A.1 macrophages. In C6 astrocytes, L-NAME gave a percentage of inhibition of 39.33 ± 4.37% and 46.21 ± 2.65% of nitrite production and iNOS expression, respectively. NAC (10 mM) was used as positive control for ROS release and gave a percentage of inhibition of ROS production of 34.50 ± 0.50% in J774A.1 macrophages and 43.33 ± 1.45% in C6 astrocytes.

The phytochemical investigation of the dichloromethane extract revealed three tremetone-derivatives: 6,12-dihydroxy-(−)-2*S*-tremetone (**1**), (*S*)-2-(5-acetyl-6-hydroxy-2,3-dihydrobenzofuran-2-yl)allyl isobutyrate (**2**) and 2-((*S*)-5-acetyl-6-hydroxy-2,3-dihydrobenzofuran-2-yl)allyl (*S*)-2-methylbutanoate (**3**). The evaluation of those compounds for their anti-inflammatory and anti-oxidant potential indicate a significant inhibitory activity at all tested concentrations (5–50 µM) both on NO release, iNOS expression, and ROS production, and in both J774A.1 (Figure 6A–C) and C6 cells (Figure 6D–F). In particular, 6,12-dihydroxy-(−)-2*S*-tremetone (**1**) showed the highest activity in the performed experiments and reduced the ROS production in macrophages at 10 µM by approximately 50% while in C6 cells a concentration of approximately 45 µM was needed for the same reduction. Comparable results were observed for the measured NO-release, where ca. 15 µM are needed for 50% reduction of NO-release in macrophages and ca. 25 µM for the same reduction in C6 cells and for measured iNOS expression, where ca. <5 µM are needed for 50% reduction of iNOS-expression in macrophages and ca. <5 µM for the same reduction in C6 astrocytes.

In our opinion, the compound class of benzofurans seems to be a promising substance group for further investigations, although some related derivatives are suspected to be harmful. Those compounds, tremetone, 6-hydroxy-trementone, and further derivatives, can be found in white snake-root (*Ageratina altissima* syn. *Eupatorium rugosum*, Asteraceae) and are suspected to cause trembles in livestock and the so-called “milk sickness syndrome” in humans. A recent in vivo study in goats [20] investigated, beside other preparations, the in vivo effect (intra-ruminal application) of an *n*-hexane extract containing 2.10 μg/mg, 0.96 μg/mg, and 0.53 μg/mg of tremetone, 6-hydroxytremetone, and dehydrotremetone. The extract group showed no clinical signs, exercise intolerance, serum enzyme changes, or histological lesions as observed for the snake-root plant dosed group. Therefore, the authors concluded that there may be another compound or group of compounds that act either independently or synergistically with tremetone, or other benzofurans, to cause the myotoxic effect of white snake-root in goats. This study supports the investigations of Bowen et al., who showed that tremetone is non-toxic (short term, 7d) to mice, rabbits, lambs, or chickens, while the compound affected goldfish and insects [21]. In accordance with these results, our experiments showed, in the investigated time interval and dosages, no signs of toxic effects in the performed MTT assay, which showed no effect on macrophages and astrocytes proliferation (extract and by tested compounds; data not shown). Therefore, the potential anti-inflammatory and anti-oxidant potential can not be ascribed to an effect on cell viability.

## 3. Materials and Methods

### 3.1. Reagents

Unless stated otherwise, all reagents and compounds were purchased from Sigma Chemicals Company (Sigma, Milan, Italy).

### 3.2. Cell Cultures

#### 3.2.1. J774A.1 Murine Macrophages Cell Line

J774A.1 murine monocyte macrophage cell line (American Type Culture Collection, Rockville, MD, USA), was grown adjacent to Petri dishes with Dulbecco’s modified Eagle’s medium (DMEM), supplemented with 10% fetal calf serum (FCS), 25 mM HEPES, 2 mM glutamine, 100 U/mL penicillin, and 100 mg/mL streptomycin at 37 °C in a 5% CO_2_ atmosphere. Macrophages J774A.1 were plated in 96 well plates (2.0 × 10^4^) and allowed to adhere for 4 h. Thereafter, the medium was replaced with pure fresh medium or containing serial dilutions of *D. austriacum* root extracts (5–50 μg/mL) or isolated compounds **1**–**3** (5–50 μM) for 1 h, and then co-exposed to a final concentration of LPS (1 μg/mL) for further 18 or 24 h for NO, and ROS detection. L-NAME (1 µM) and NAC (10 mM) were used as positive controls.

#### 3.2.2. C6 Astroglial Cell Line

Glioma cells (C6) were obtained from American Type Culture Collection (ATCC, Manassas, VA, USA). Cells were grown in DMEM, 10% FBS (mL/L), 2 mM l-glutamine and penicillin/streptomycin (100 units/0.1 mg/mL) at 37 °C in 5% CO_2_ atmosphere. Cells were passaged at confluence using a solution of 0.025% trypsin and 0.01% EDTA. C6 astrocytes were plated in 96 well plates (2.0 × 10^4^), and allowed to adhere for 24 h. Thereafter, the medium was replaced with fresh medium alone or containing serial dilutions of isolated compounds **1**–**3** (5–50 μM) for 1 h, and then co-exposed to a final concentration of LPS (1 μg/mL) plus Interferon-y (IFN 100 U/mL) for further 24 h in order to evaluate NO and ROS release. L-NAME (1 µM) and NAC (10 mM) were used as positive controls.

#### 3.2.3. Antiproliferative Assay

J774A.1 macrophages (5 × 10^4^/well) and C6 cells (2.0 × 10^4^/well) were plated on 96-well plates and allowed to adhere for 4 h and 24 h, respectively. Thereafter, the medium was replaced with fresh medium alone or containing serial dilutions of the extract or of the isolated compounds **1**–**3** (5–50 μg/mL and 5–50 μM respectively), and incubation was performed for 24, 48 and 72 h. Cell viability was assessed through 3-(4,5-dimethyltiazol-2yl)-2,5-phenyl-2*H*-tetrazolium bromide (MTT) assay as previously reported [22]. Briefly, 25 μL of MTT (5 mg/mL) was added and the cells were incubated for an additional 3 h. Thereafter, cells were lysed and the dark blue crystals solubilized with 100 μL of a solution containing 50% (mL/L) *N*,*N*-dimethylformamide, 20% (mL/L) sodium dodecyl sulfate (SDS) with an adjusted pH of 4.5. The optical density (OD) of each well was measured with a microplate spectrophotometer Titertek (Dasit, Cornaredo, Milan, Italy), equipped with a 620 nm filter. C6 viability in response to treatment was calculated as: % dead cells = 100 − (OD treated/OD control) × 100.

#### 3.2.4. Measurement of Intracellular ROS

ROS formation was evaluated by means of the probe 2′,7′-dichlorofluorescin-diacetate (H2DCF-DA). H2DCF-DA is a non-fluorescent permeant molecule that passively diffuses into cells, where the acetates are cleaved by intracellular esterases to form H2DCF, and thereby traps it within the cell. In the presence of intracellular ROS, H2DCF is rapidly oxidized to the highly fluorescent 2′,7′-dichlorofluorescein (DCF). After cell treatment, previously reported, cells were collected, washed twice with phosphate buffer saline (PBS), and then incubated in PBS containing H2DCF-DA (10 µM) at 37 °C. After 15 min, fluorescence was evaluated using a fluorescence-activated cell sorting (BD FacsCalibur, Milan, Italy) and elaborated with Cell Quest software, as previously reported [23].

#### 3.2.5. Measurement of NO Release

NO generation was measured as nitrite (NO_2_^−^), index of NO released by cells, in the culture medium of J774A.1 macrophages and C6 astrocytes 24 h after LPS, or LPS + IFN-γ, stimulation by Griess reaction, as previously reported [24]. Briefly, 100 mL of cell culture medium were mixed with 100 mL of Griess reagent, equal volumes of 1% (*w*:*v*) sulphanilamide in 5% (*v*:*v*) phosphoric acid and 0.1% (*w*:*v*) naphtylethylenediamine–hydrogen chloride and incubated at room temperature for 10 min, and then the absorbance was measured at 550 nm in a microplate reader Titertek (Dasit, Cornaredo, Milan, Italy). The amount of NO_2_^−^ in the measured samples is expressed as μM concentration, which was calculated via a sodium NO_2_^−^ standard curve.

#### 3.2.6. iNOS Detection by Cytofluorimetry

J774A.1 macrophages and C6 astrocytes were plated into 96-well plates (5 × 10^4^ cells/well) and were allowed to grow for 24 h at 37 °C in a 5% CO_2_ atmosphere before experiments. Thereafter, the medium was replaced with fresh medium, and cells were treated with serial dilutions of the extract (5–50 μg/mL) or of the isolated compounds (5–50 μM) added 1 h before and simultaneously to LPS or LPS + IFN-γ, respectively, for 24 h. Cells were collected, washed twice with PBS, incubated in Fixing Solution for 20 min at 4 °C, and then incubated in Fix Perm Solution for 30 min at 4 °C. Anti-iNOS (BD Laboratories, Milan, Italy) was added to J774A.1 and C6 cells for a further 30 min. The secondary antibody was added in Fix Perm solution and cells were evaluated using a fluorescence-activated cell sorting (FACSscan; Becton Dickinson, Milan, Italy) and elaborated with Cell Quest software, as previously reported [25].

#### 3.2.7. Nitrotyrosine Detection by Confocal Microscopy

J774A.1 macrophages and C6 cells (3 × 10^5^/well) were seeded on coverslips in 12 well plate and treated with serial dilutions of the extract or of the isolated compounds (5–50 μg/mL and 5–50 μM respectively) added 1 h before and simultaneously to LPS or LPS + IFN-γ for 24 h. Then, cells were fixed with 4% paraformaldehyde in PBS for 15 min and permeabilized with 0.1% saponin (Sigma #47036) in PBS for 15 min. After addition of BSA and PBS and a waiting time of 1 h, cells were incubated with rabbit anti-nitrotyrosine (Millipore, Billerica, MA, USA) for 1 h at room temperature. The slides were then washed with PBS three times and fluorescein-conjugated secondary antibody (FITC) was added and left for 1 h. DAPI was used for counterstaining of nuclei. Coverslips were finally mounted in mounting medium and fluorescent images were taken under the Laser Confocal Microscope (Leica TCS SP5) as previously reported [26].

#### 3.2.8. Data Analysis

Data are reported as mean ± standard error of the mean (S.E.M.) values of at least three independent experiments. Statistical analysis was performed by analysis of variance test, and multiple comparisons were made by Bonferroni’s test by using Prism 6 (GraphPad Software, San Diego, CA, USA). *p*-values smaller than 0.05 were considered significant.

#### 3.2.9. Plant Material

The plant material used in this study was collected at two locations close to Wels, Austria (herbarium number, Pharmacognosy, University of Innsbruck: MD 00-1848, 282.0 g; MD-2050, 254.0 g). The plants were in flowering stage and were identified by Dr. Michael Dobner. In order to obtain a higher amount of starting material, both batches were combined and processed together.

#### 3.2.10. Extract Preparation

The milled plant material (536.0 g) was mixed with 2 L dichloromethane and macerated at room temperature for 24 h. Before further processing, the mixture was sonicated for 10 min and filtered. The remaining plant material was further extracted as described before (in total five times), and the combined solutions were evaporated to dryness to yield 10.02 g of DCM-extract.

#### 3.2.11. Isolation

A part of the obtained DCM-extract (7.17 g) was separated by Sephadex-LH20 CC (GE Healthcare, Uppsala, Sweden; 1.5 cm × 80 cm) using DCM/acetone (85/15; *v*/*v*) as mobile phase. Eluate was collected in tubes in portions of 8 mL, which were combined according to their composition indicated by TLC (Merck, Darmstadt, Germany, silica gel 60 on aluminum, layer 0.2 mm, F_254_; No. 5554; mobile phase: dichloromethane + ethyl acetate + acetic acid (5 + 0.5 + 0.1; *v*/*v*/*v*) at UV 366 nm. Fraction 3 (tubes 35 to 49, 3.93 g) was further separated by silica gel CC (silica gel 60; 40–63 µm, Merck, Darmstadt, Germany; 100 g, 3 × 45 cm) using DCM/acetone mixtures with small amounts of acetone (<2.5 *v* %) for elution. The fraction eluted with 610–790 mL (421.91 mg) was further separated by semi-preparative HPLC (Dionex-system, UltiMate 3000, Dionex Softron GmbH, Germering, Germany); stationary phase: Phenomenex Aqua C18 column (5 µm; 250 × 10 mm); mobile phase: 80% methanol, 20% water, isocratic; flow 2.00 mL/min; 40 °C; 50 µL injection volume of a solution of 52.74 mg/mL methanol). Separation of 4.5 mL afforded **2** (R_t_: 17.56–18.35 min; 88.64 mg; (*S*)-2-(5-acetyl-6-hydroxy-2,3-dihydrobenzofuran-2-yl)allyl isobutyrate and **3** (R_t_: 21.66–22.51 min; 39.20 mg; 2-((*S*)-5-acetyl-6-hydroxy-2,3-dihydrobenzofuran-2-yl)allyl (*S*)-2-methylbutanoate. Fraction 5 of the initial Sephadex CC (tube 67–120; 334.64 mg) was further separated by silica gel CC (4.3 g, 1.2 × 43 cm) using DCM/acetone mixtures with increasing amounts of acetone. A fraction eluted with 300–980 mL (53.89 mg) was further purified by semi-preparative HPLC (stationary phase: Phenomenex Aqua C18 column (5 µm; 250 × 10 mm); mobile phase: solvent A: water solvent B: acetonitrile, gradient elution: 0 min: 45% A; 16 min: 2% A; 21 min: 45% A; 30 min: stop; flow 2.00 mL/min; 40 °C; 150 µL injection volume of a solution of 18.00 mg/mL methanol). The collection of the eluate at R_t_ 12.00–13.89 min of 20 injections afforded 26.51 mg of compound **1**, 6,12-dihydroxy-(−)-2*S*-tremetone.

HPLC-MS analyses were carried out on a 1100 Agilent system (Agilent, Waldbronn, Germany) using the following analytical conditions: stationary phase: Phenomenex Synergi Max-RP 80 Å, 4 µm (4.6 mm × 150 mm); temperature: 35 °C; mobile phase: A = water + 0.9% formic acid and 0.1% acetic acid, B = acetonitrile; flow rate: 1.00 mL/min; detection: 205 nm; injection volume: 10 µL; solvent composition during analysis:0′: 75% A; 10′: 60% A; 30′: 30% A; 35′: 2% A; 45′: stop; posttime 10′. HPLC was hyphenated via 1:5 split to an Esquire 3000 plus (Bruker Daltonics, Bremen, Germany) ion trap using ESI in alternating mode with the following settings: temperature: 350 °C; dry gas: 10.00 L/min; nebulizer 40 psi N_2_; full scan mode: *m*/*z* 100–1500. The optical rotations were determined with a Perkin-Elmer 341 polarimeter (Wellesley, MA, USA) at 20 °C. 1D- and 2D-NMR-experiments were performed on a Bruker DRX 300 (Bruker Biospin Rheinstetten, Germany) operating at 300.13 MHz (^1^H) and 75.48 MHz (^13^C) at 300 K; NMR solvent: CDCl_3_ with 0.03% TMS (Eurisotop, Gif-Sur-Yvette, France), which was used as an internal standard. ECD spectra were simulated using the 3D structure of **1** which was forwarded to Schrödinger MacroModel 9.1 (Schrödinger LLC., New York, NY, USA) to perform conformational analysis under OPLS-3 molecular mechanic force field in H_2_O. Conformers in an energy window of 2 Kcal/mol distance from global minima were subjected to geometrical optimization and energy calculation using DFT with B3LYP/6-31G** level of theory in gas phase with Gaussian 09 (Gaussian 09, Revision A.02, Gaussian, Inc., Wallingford, CT, USA, 2016). Calculation of excitation energy (nm), rotatory strength dipole velocity (R*_vel_*) and dipole length (R*_len_*) were performed by TDDFT/CAM-b3LYP/6-31G** in acetonitrile using SCRF (self-consistent reaction field) method with the CPCM (conductor-like polarizable continuum). ECD curves were simulated on the basis of rotatory strengths using SpecDis v1.6 [27] with half-band of 0.25 eV. ECD spectra were recorded in acetonitrile at a Jasco J715 instrument (Easton, MD, USA).

For the hydrolysis experiment, 2.75 mg of compound **3** was dissolved in 0.50 mL of 3 M aqueous trifluoroacetic acid and heated in a closed glass container for 2 h to 90 °C. Then, the cooled hydrolysate was mixed three times with 1.0 mL of diethyl ether to recover the free alcohol part. The combined organic layer was evaporated to dryness (yield: 2.01 mg, 99.3% of theoretical yield) and optical rotation was measured (${\left[\alpha \right]}_{20{}^{\circ}C}^{578\;\mathrm{nm}}$: −32.83; *c* 1.005, MeOH); while the remaining water fraction was filled up to 2.00 mL with 3 M aqueous trifluoroacetic acid and measured directly (measured value +0.015°; specific rotation, calculation based on the yield of the alcohol part: ${\left[\alpha \right]}_{20{}^{\circ}C}^{578\;\mathrm{nm}}$: +34.25; *c* 0.044, 3 M aqueous trifluoroacetic acid).

## 4. Conclusions

This study provides evidence for the pharmacological anti-inflammatory and anti-oxidant potential of *Doronicum austriacum* extract. Phytochemical investigations identified the three major compounds of the DCM root extract as 6,12-dihydroxy-(−)-2*S*-tremetone, (*S*)-2-(5-acetyl-6-hydroxy-2,3-dihydrobenzofuran-2-yl)allyl isobutyrate , and 2-((*S*)-5-acetyl-6-hydroxy-2,3-dihydrobenzofuran-2-yl)allyl (*S*)-2-methylbutanoate, which were analyzed the first time for their ability to lower NO and ROS release. Termetone derivatives were identified as a promising compound class to combat inflammation and related diseases.

## Figures and Tables

**Table 1:** ^1^H (300.13 MHz) and ^13^C (75.48 MHz) NMR data of compounds **1** to **3** (in CDCl_3_, referenced to 0.01% TMS; δ in ppm; multiplicity; *J* in Hz in parenthesis).

Position	^1^H-NMR Data of 1	^13^C-NMR Data of 1	^1^H-NMR Data of 2	^13^C-NMR Data of 2	^1^H-NMR Data of 3	^13^C-NMR Data of 3
2	5.40 *t* (8.6)	85.3 *d*	5.36 *t* (8.4)	84.9 *d*	5.36 *t* (8.4)	84.9 *d*
3	H_a_ 3.36 *ddd* (0.9, 9.4, 15.4) H_b_ 3.09 *ddd* (1.3, 7.8, 15.3)	33.6 *t*	H_a_ 3.36 *ddd* (0.8, 9.5, 15.2) H_b_ 3.10 *ddd* (1.1, 7.5, 15.3)	33.6 *t*	H_a_ 3.35 *ddd* (0.8, 9.5, 15.2) H_b_ 3.10 *ddd* (1.2, 7.6, 15.4)	33.7 *t*
3a	---	118.5 *s*	---	118.3 *s*	---	118.7 *s*
4	7.49 *t* (1.3)	126.9 *d*	7.50 *t* (1.3)	126.7 *d*	7.49 *t* (1.2)	126.7 *d*
5	---	113.8 *s*	---	113.9 s	---	114.0 *s*
6	---	165.3 ^1^ *s*	---	165.6 *s*	---	166.1 *s*
7	6.36 *s*	98.3 *d*	6.37 *s*	98.3 *d*	6.37 *s*	98.3 *d*
7a	---	165.3 ^1^ *s*	---	166.0 *s*	---	166.5 *s*
8	---	201.9 *s*	---	202.0 *s*	---	202.1 *s*
9	2.53 *s*	26.1 *q*	2.53 *s*	26.2 *q*	2.53 *s*	26.1 *q*
10	---	146.8 *s*	---	142.5 *s*	---	142.6 *s*
11	H_a_ 5.27 *dq* (2.1, 0.7) H_b_ 5.25 *dt* (1.7, 0.9)	112.8 *t*	H_a_ 5.33 *br s* H_b_ 5.27 *br s*	114.6 *t*	H_a_ 5.33 *br s* H_b_ 5.28 *br s*	114.8 *t*
12	4.25 *s*	62.8 *t*	4.67 *dd* (6.7, 13.6)	63.4 *t*	4.67 *dd* (7.8, 13.5)	63.2 *t*
OH at C_6_	12.96 *s*	---	12.95 *s*	---	12.95 *s*	---
1’			---	176.8 *s*	---	176.0 *s*
2’			2.56 *hept* (7.0)	33.9 *d*	2.38 *hex* (7.0)	41.0 *d*
3’			1.16 *d* (7.0)	18.7 *q*	H_a_ 1.67 *m* H_b_ 1.47 *hept* (7.0)	26.6 *t*
4’			1.16 *d* (7.0)	18.7 *q*	0.89 *t* (7.4)	11.8 *q*
5’					1.14 *d* (7.0)	16.4 *q*

^1^ Signals overlapping.

## Supplementary Materials

The following are available online: Figures S1–S17 and structures of conformers of compound **1** used for ECD calculations.
