# Supplementary material for: Anti-Inflammatory and Anti-Oxidant Potential of the Root Extract and Constituents of Doronicum austriacum
Source: Molecules. 2017 Jun 16;22(6):1003. doi: 10.3390/molecules22061003 (PMC6152664; doi:10.3390/molecules22061003)
Supplement: Supplementary file 1 [file molecules-22-01003-s001.pdf]

Supplementary Materials

Anti-inflammatory and Anti-oxidant potential of the root extract and constituents of *Doronicum austriacum*

Stefania Marzocco[1], Simona Adesso[1 #], Mostafa Alilou[2], Hermann Stuppner[2] and Stefan Schwaiger[2,*]

[1] Department of Pharmacy, University of Salerno, Via Giovanni Paolo II 132, I-84084, Fisciano, Salerno, Italy; smarzocco@unisa.it

[#] PhD Program in Drug Discovery and Development, University of Salerno, Via Giovanni Paolo II 132, I-84084 Fisciano, SA, Italy; sadesso@unisa.it

[2] Institute of Pharmacy, Pharmacognosy, Member of the CMBI, University of Innsbruck, CCB, Innrain 80-82, 6020 Innsbruck; stefan.schwaiger@uibk.ac.at; hermann.stuppner@uibk.ac.at; mostafa.alilou@student.uibk.ac.at

* Correspondence: stefan.schwaiger@uibk.ac.at; Tel.: +43-512-507-58409

Spectra of compound 1

Figure S1: LC-MS-spectrum (positive Mode)

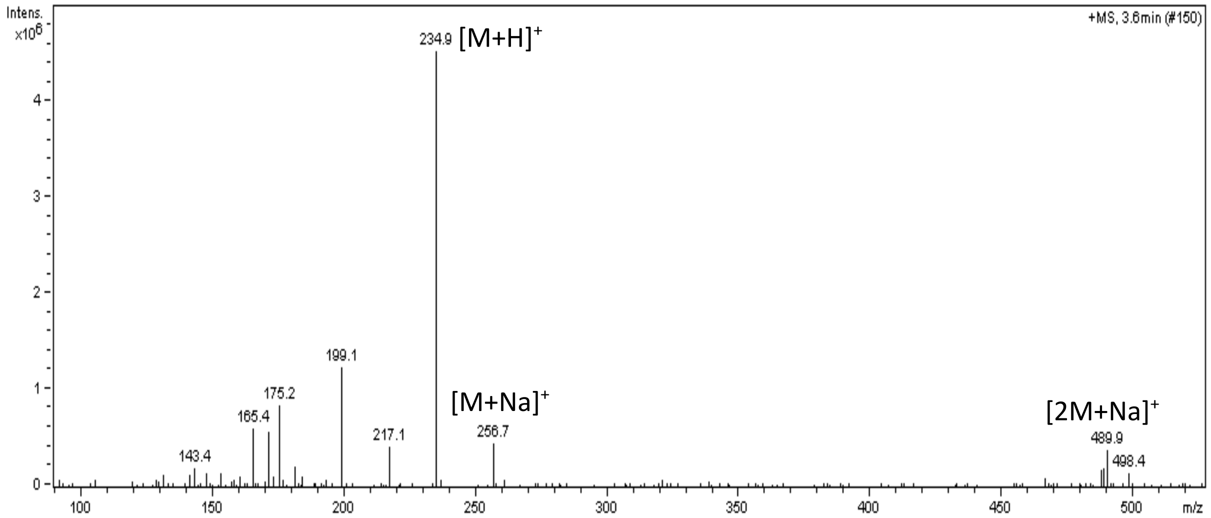

Figure S2: ^{1}H-NMR spectrum (300.13 MHz) of compound **1** in $CDCl_3$.

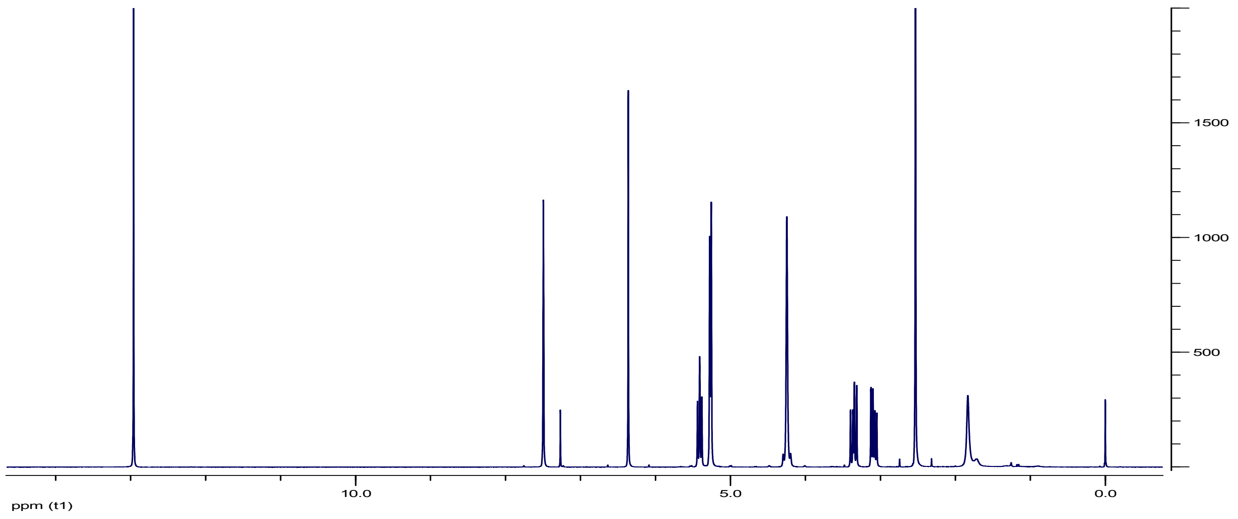

Figure S3: HSQC-NMR spectrum (300.13 MHz; 75.48 MHz) of compound **1** in $CDCl_3$.

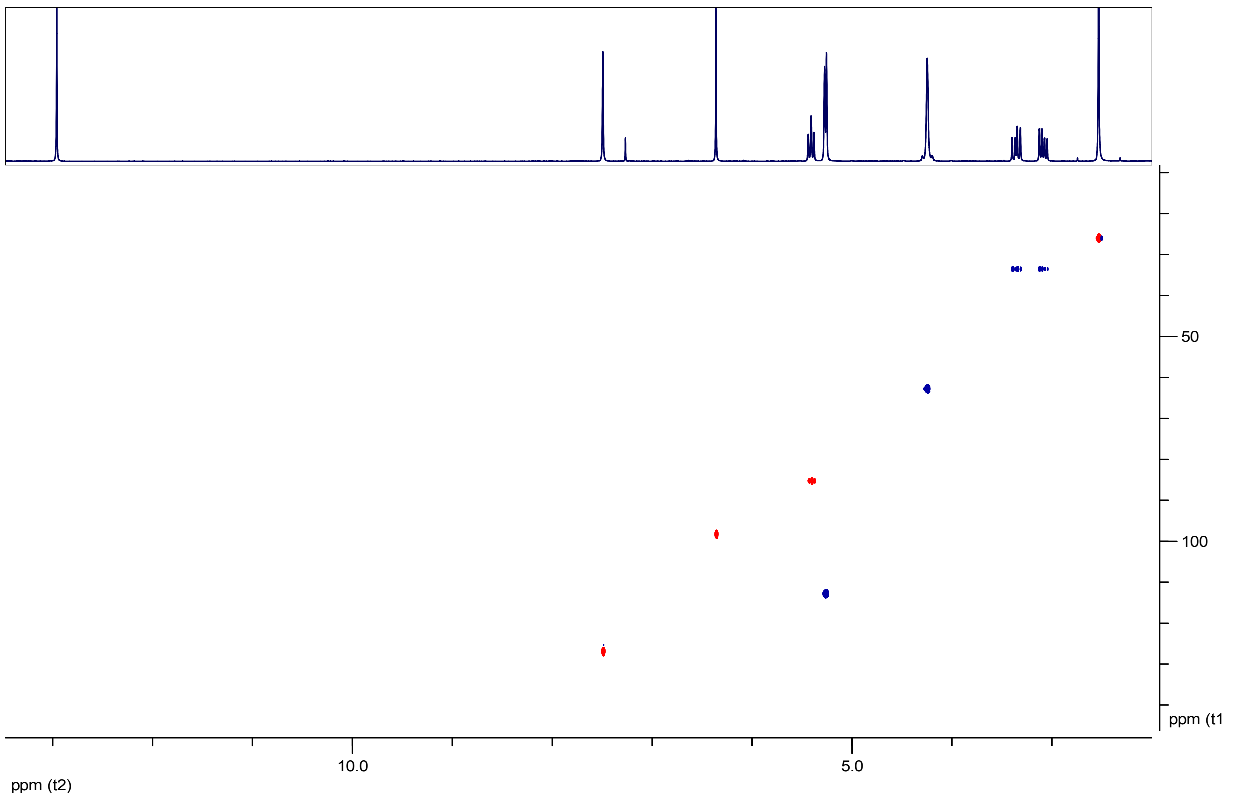

Figure S4: HMBC-NMR spectrum (300.13 MHz; 75.48 MHz) of compound **1** in $CDCl_3$.

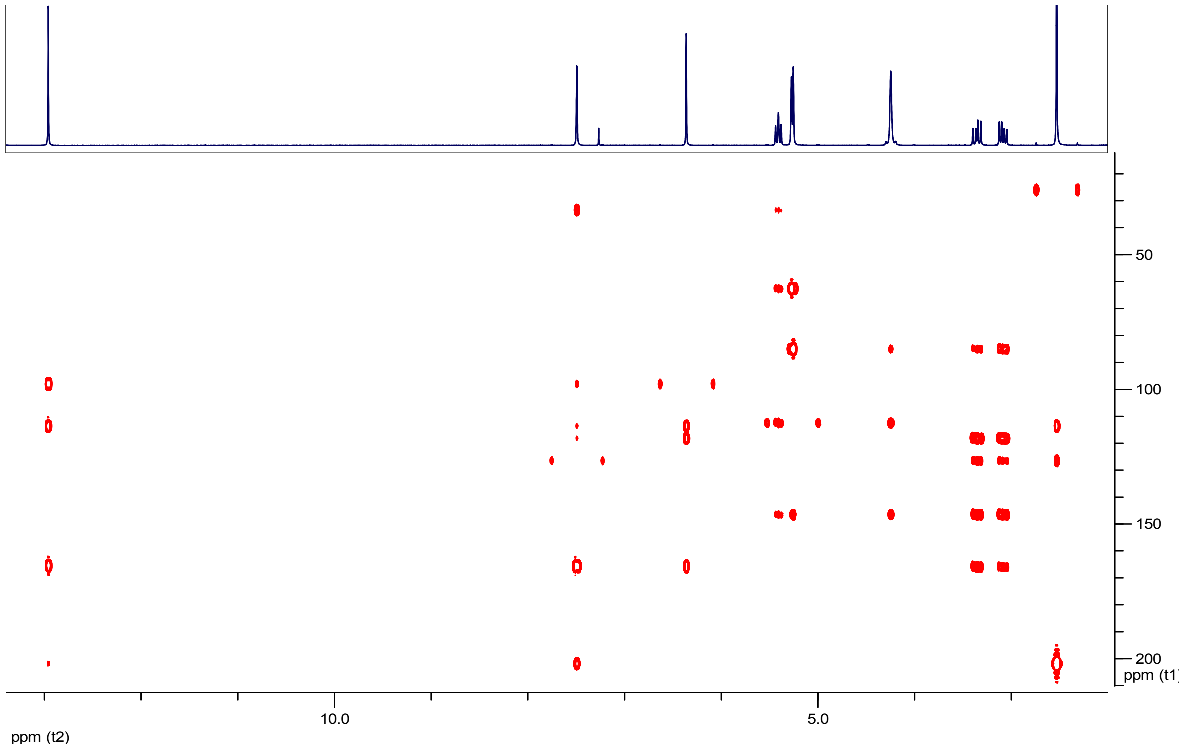

Figure S5: COSY-NMR spectrum (300.13 MHz) of compound **1** in $CDCl_3$.

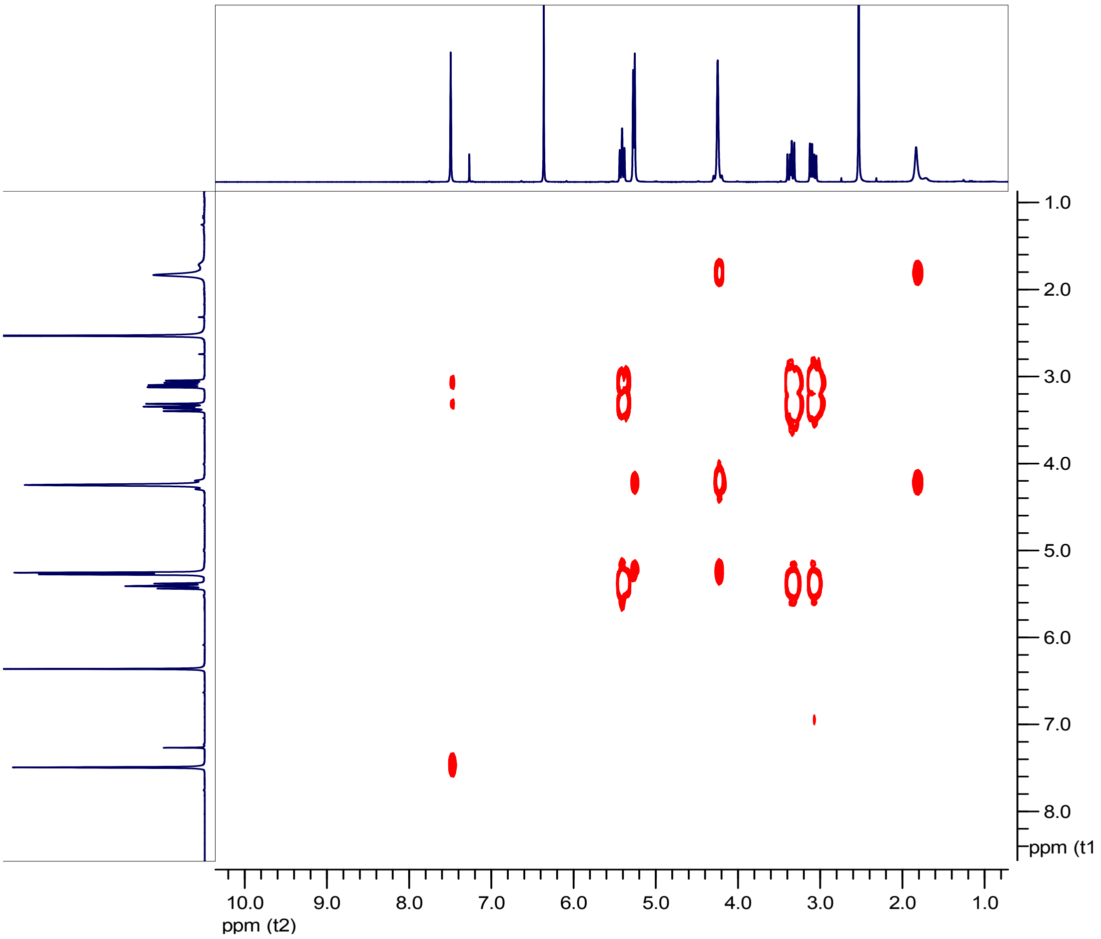

Spectra of compound 2

Figure S6: LC-MS-spectrum (positive Mode)

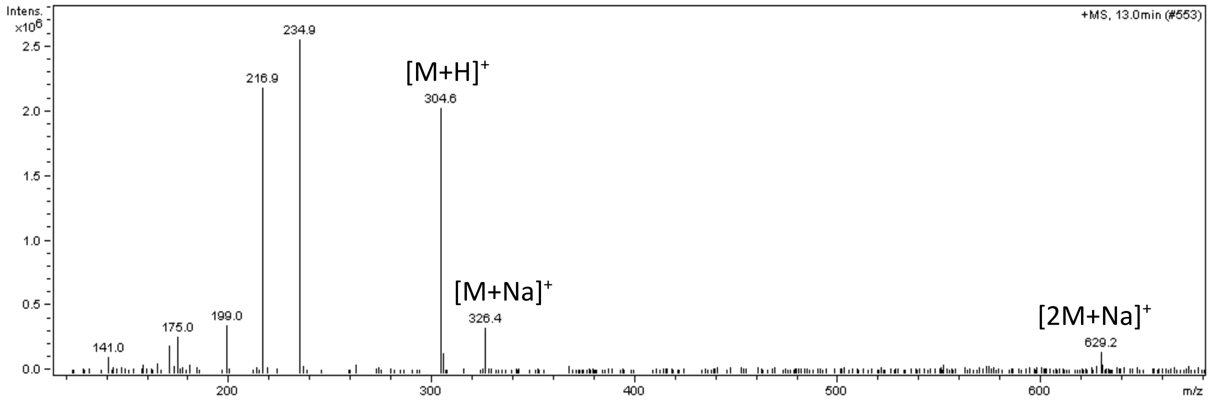

Figure S7: ^{1}H-NMR spectrum (300.13 MHz) of compound **2** in $CDCl_3$.

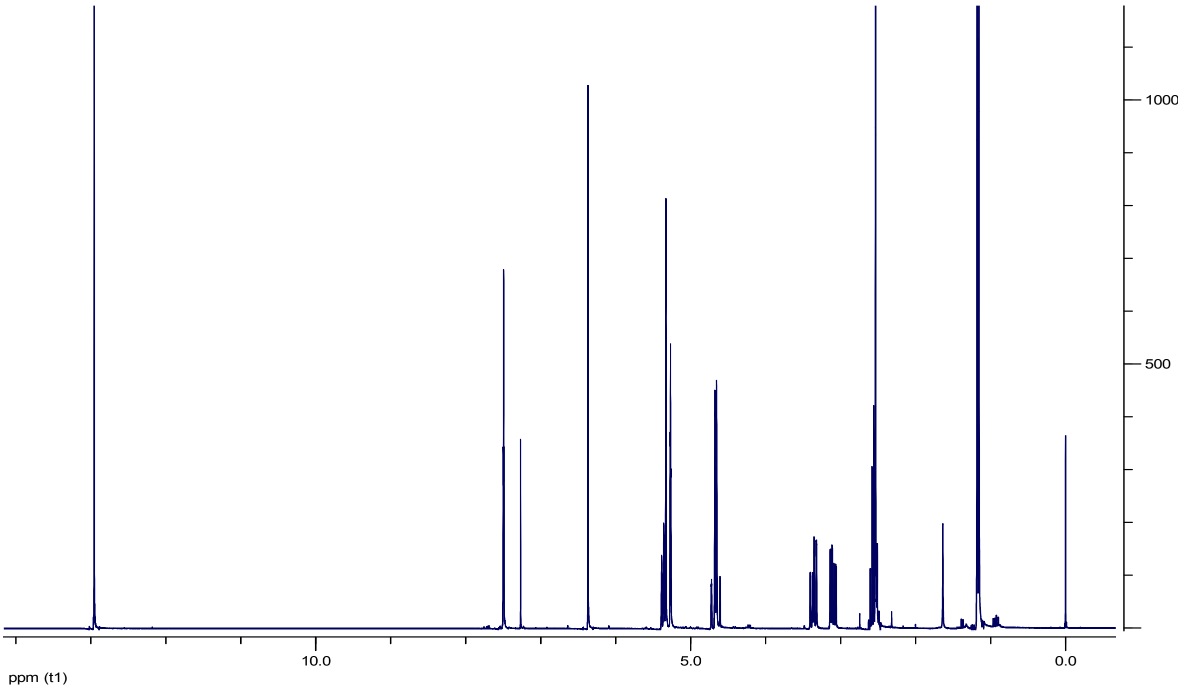

Figure S8**:** HSQC-NMR spectrum (300.13 MHz; 75.48 MHz) of compound **2** in $CDCl_3$.

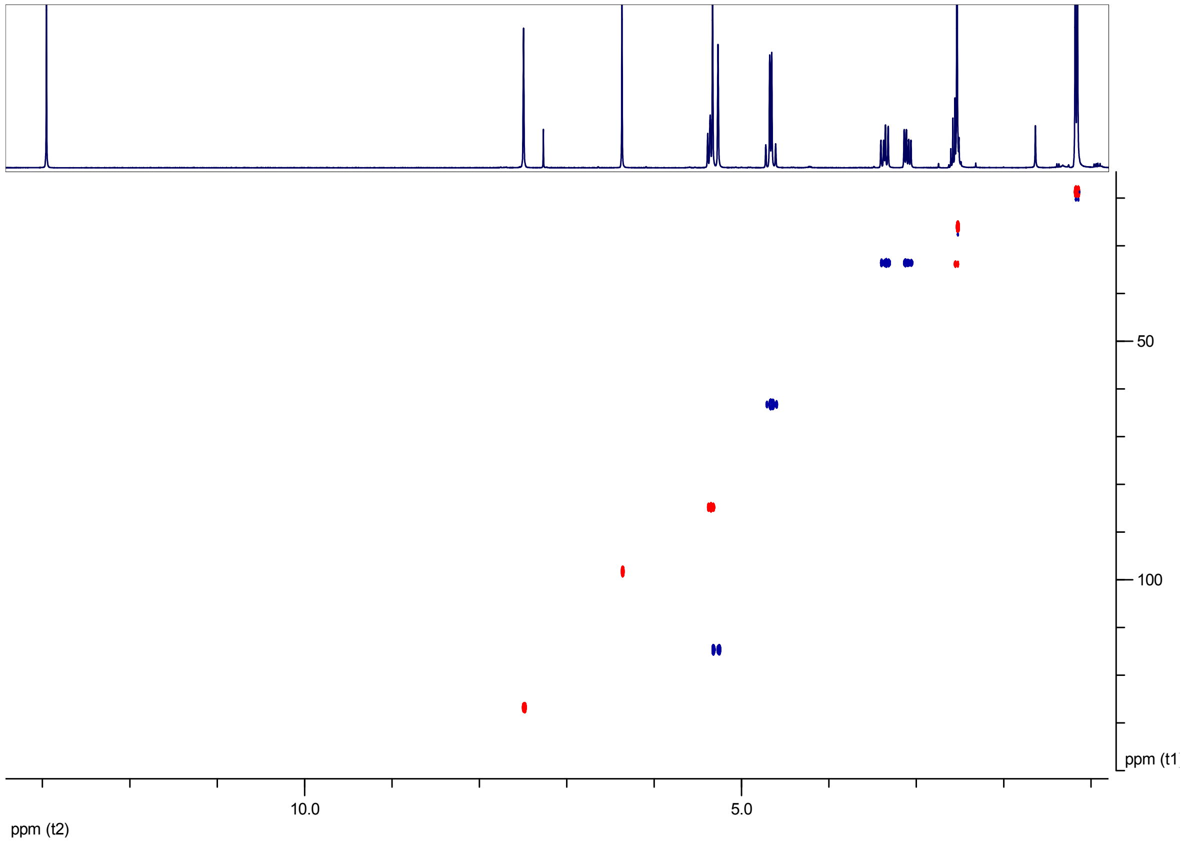

Figure S9**:** HMBC-NMR spectrum (300.13 MHz; 75.48 MHz) of compound **2** in $CDCl_3$.

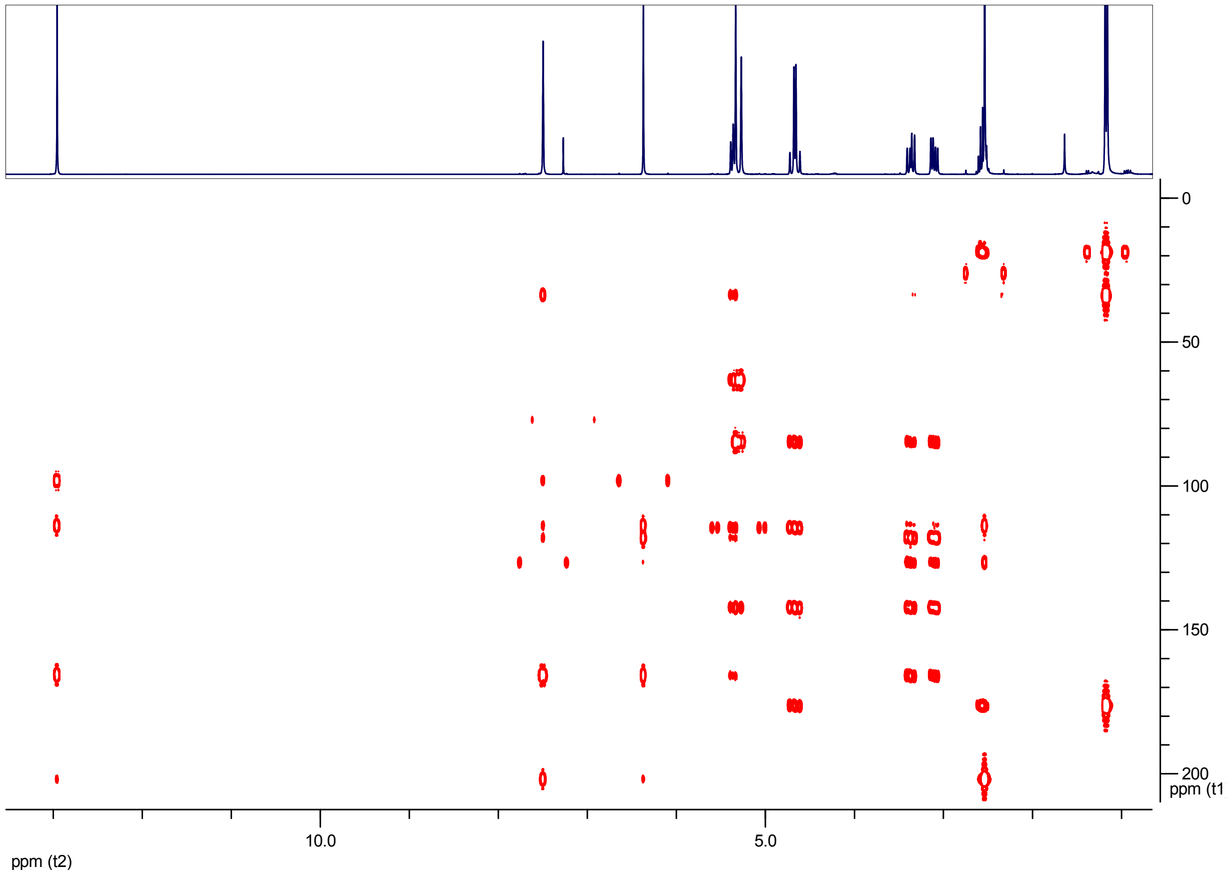

Figure S10: COSY-NMR spectrum (300.13 MHz) of compound **2** in $CDCl_3$.

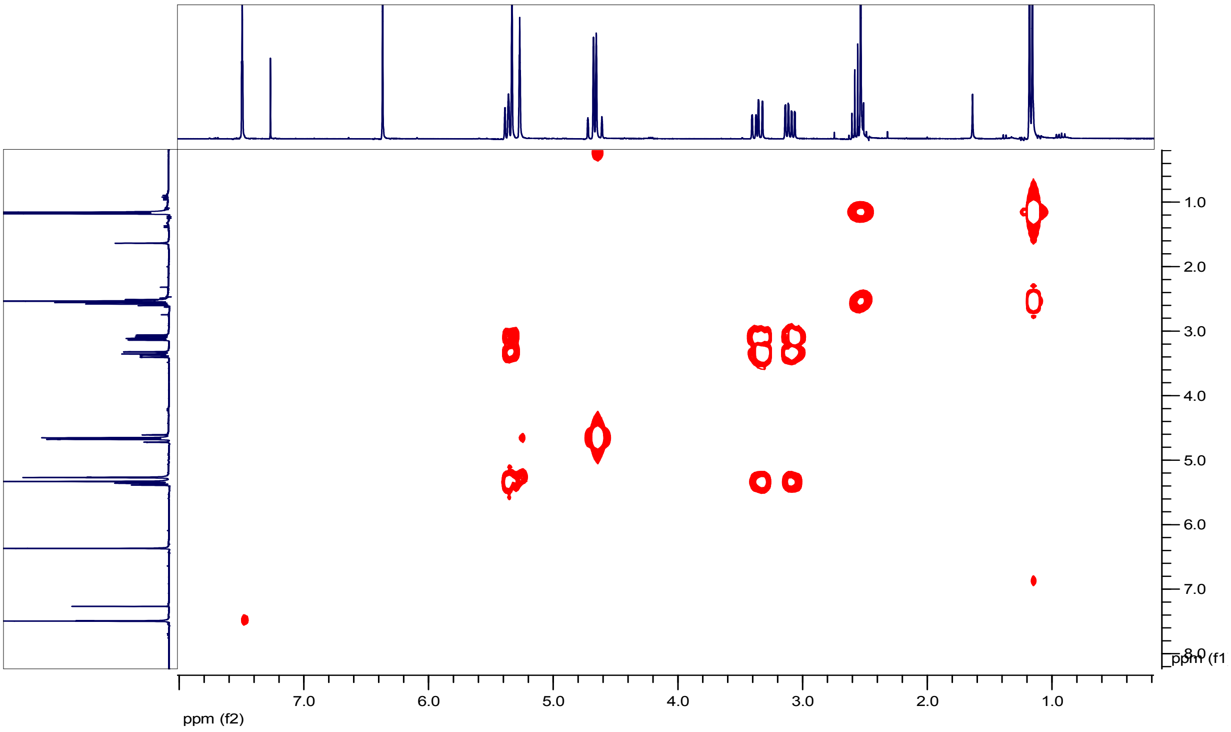

Figure S11: Experimental ECD spectrum of compound **2** in acetonitrile.

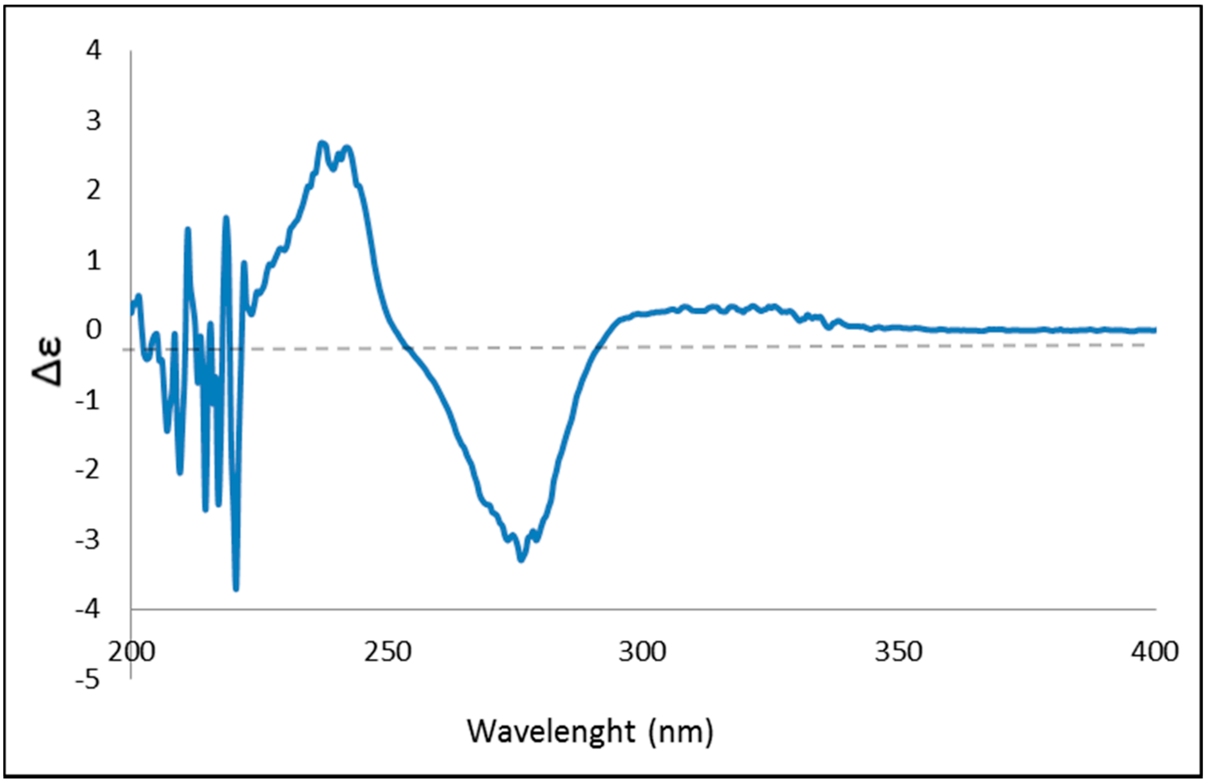

Spectra of compound 3

Figure S12: LC-MS-spectrum (positive Mode)

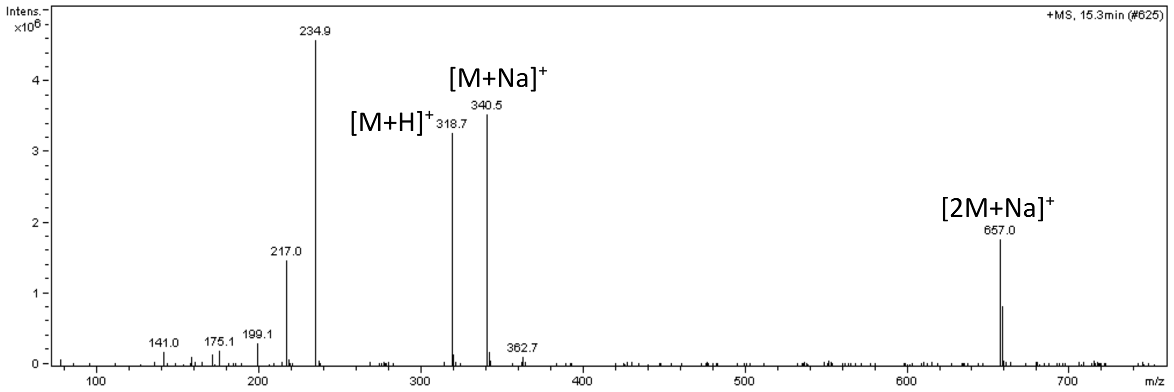

Figure S13: ^{1}H-NMR spectrum (300.13 MHz) of compound **3** in $CDCl_3$.

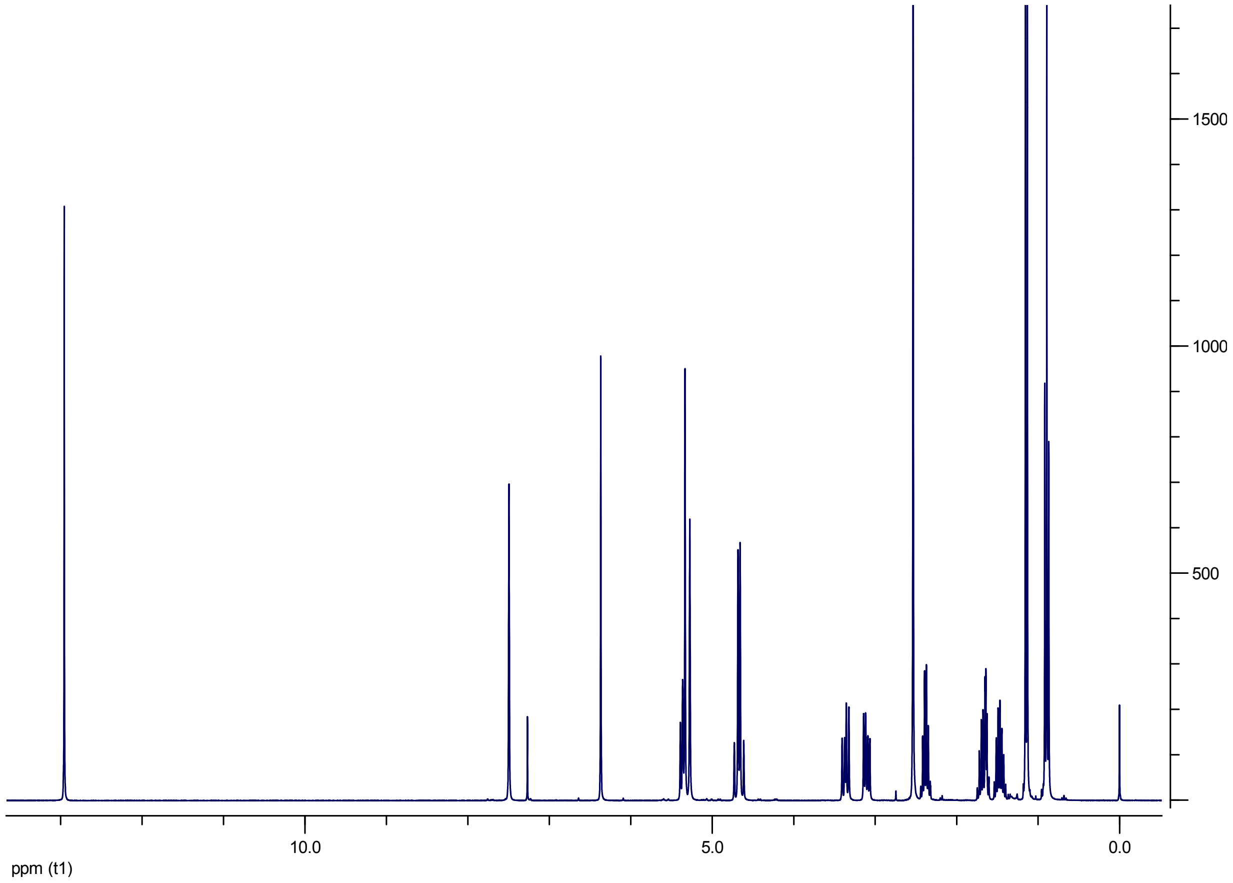

Figure S14: HSQC-NMR spectrum (300.13 MHz; 75.48 MHz) of compound **3** in $CDCl_3$.

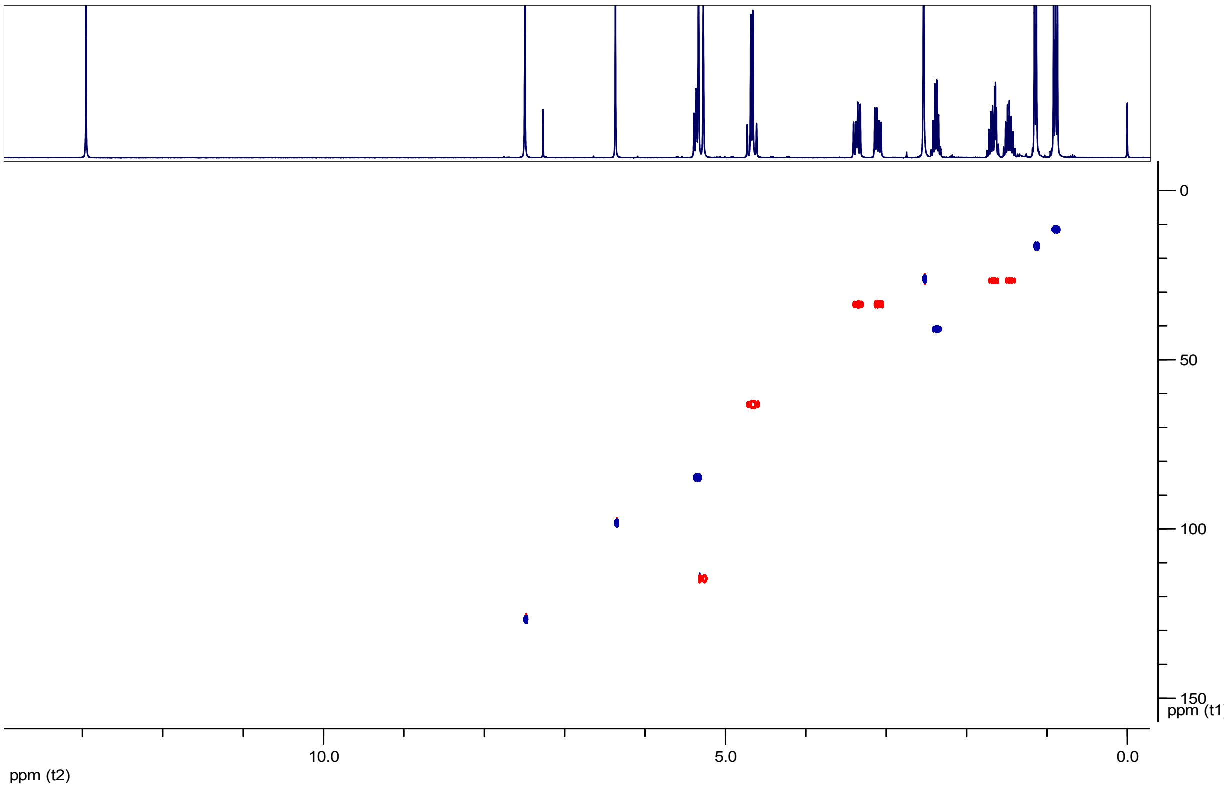

Figure S15: HMBC-NMR spectrum (300.13 MHz; 75.48 MHz) of compound **3** in $CDCl_3$.

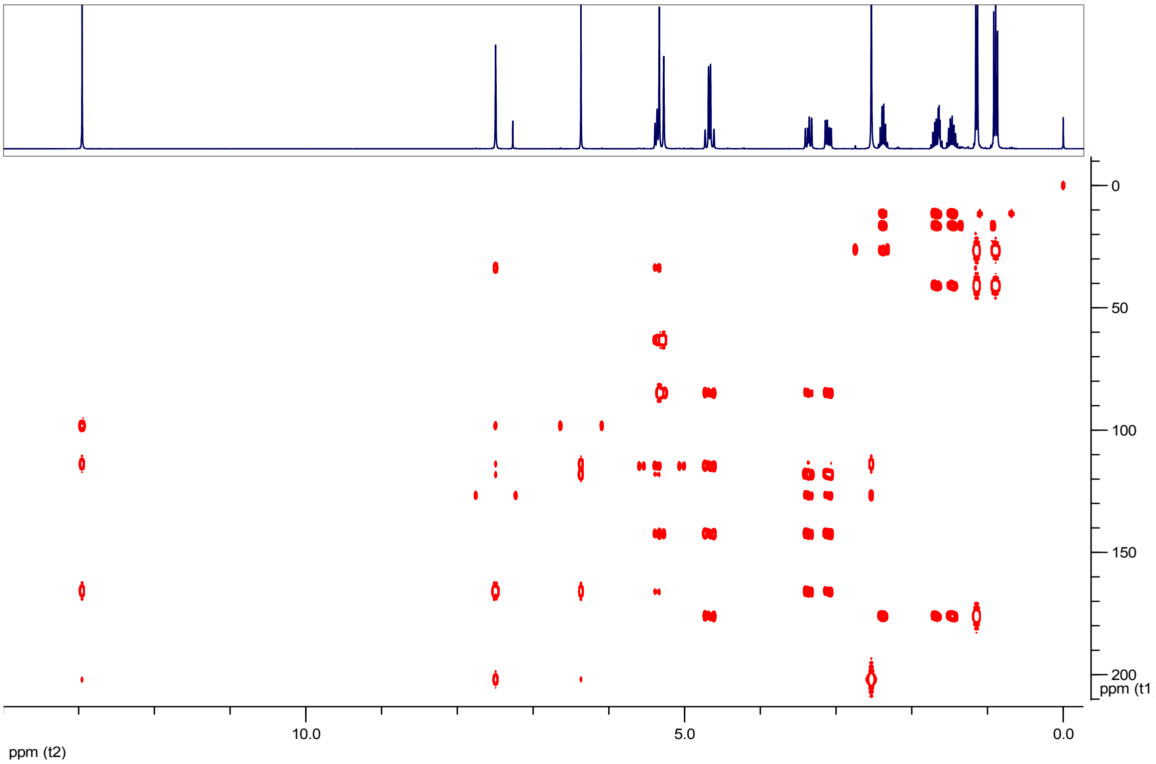

Figure S16: COSY-NMR spectrum (300.13 MHz) of compound **3** in $CDCl_3$.

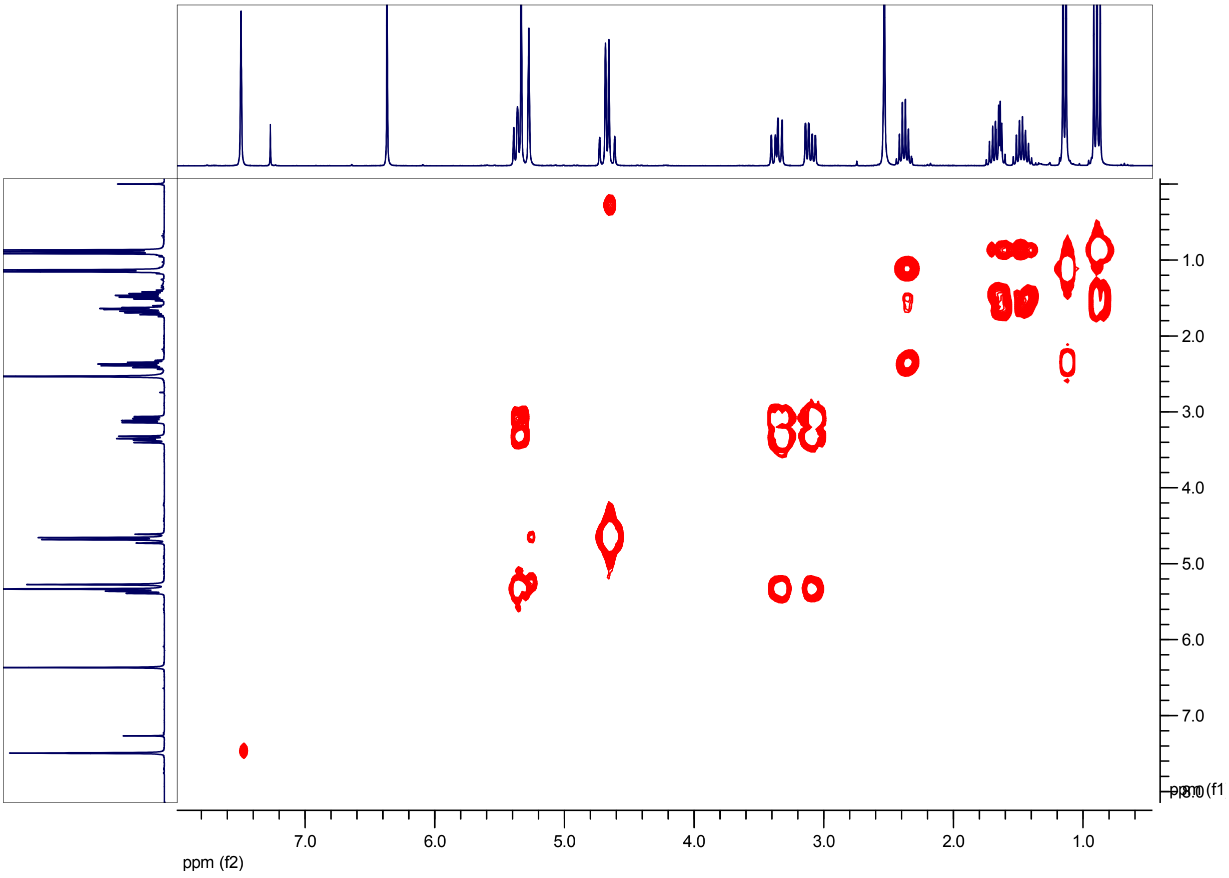

Figure S17: Experimental ECD spectrum of compound **3** in acetonitrile.

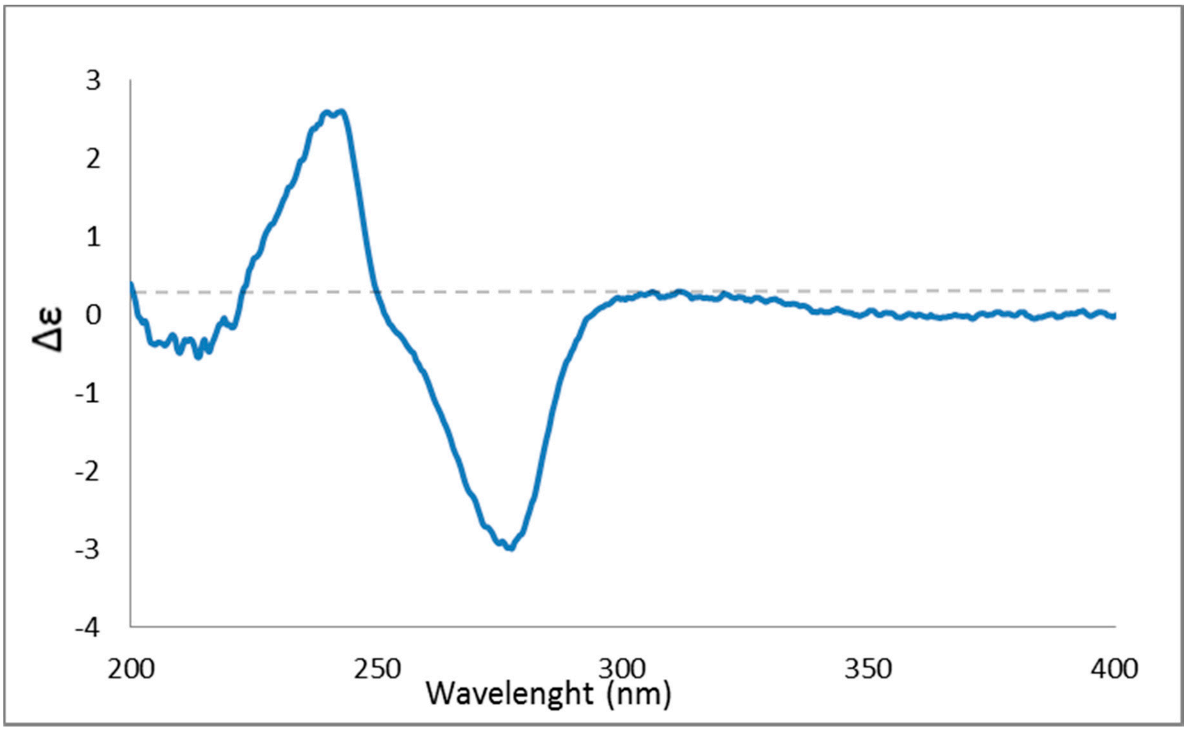

Figure S18**:** Energy minimized conformers of compound **1** using DFT at the B3LYP/6-31G** level.

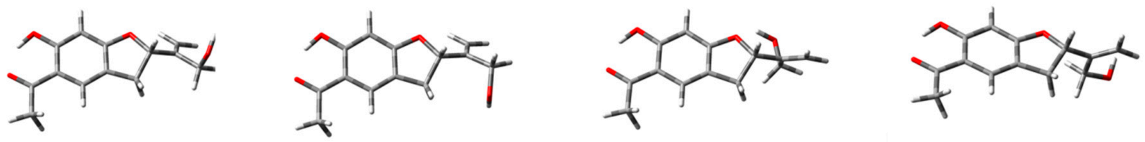

1-30.42% **2**-14% **3**-13.87% **4**-12.44%

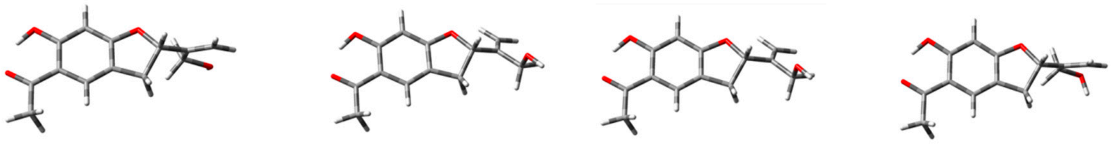

5-11.23% **6**-6.16% **7**-6.14% **8**-5.69%
